# Integration of Suicide Prevention Training into the Preclinical Medical School Curriculum

**DOI:** 10.1007/s40670-026-02637-3

**Published:** 2026-03-03

**Authors:** Riya Chhabra, Shivapriya Chandu, Ahmad Abu-Mahfouz, Kristin Sarsfield, Berkley Browne-Holtz

**Affiliations:** Oakland University William Beaumont School of Medicine, 586 Pioneer Dr, Rochester, MI 48309 USA

## Abstract

According to the Centers for Disease Control and Prevention, in 2023, 1.5 million adults attempted suicide (1). To address this mental health emergency, suicide prevention workshops have been introduced to resident physicians (5). However there exists a gap in suicide education for medical students, especially in the preclinical years. This study evaluated the feasibility and impact of integrating safeTALK, a standardized suicide prevention training, into the preclinical curriculum at Oakland University William Beaumont School of Medicine (OUWB). safeTALK was piloted as both an optional and a required component of the OUWB curriculum, and students completed pre- and post-training surveys assessing their self-perceived preparedness to identify and handle a mental health crisis. Our data shows that incorporating a brief suicide prevention program like safeTALK significantly increased students’ self-perceived abilities to both identify and handle a mental crisis. Integrating this training into the preclinical medical curriculum is both feasible and effective in improving students’ preparedness to engage with real-world mental health crises. Broader adoption of suicide prevention training in the medical school curriculum can help foster a culture of mental health awareness and equip students with life-saving intervention skills.

**Supplementary Information:**

The online version contains supplementary material available at 10.1007/s40670-026-02637-3.

## Introduction

Suicide is a pressing public health crisis. In 2023, 1.5 million adults in the United States attempted suicide, and 13.2 million had serious thoughts about it [1]. Suicide rates are also alarmingly high among healthcare professionals, with approximately 300 to 400 physicians dying by suicide each year [2]. These statistics are likely an underestimation, as stigma and fear of professional repercussions contribute to the underreporting of mental health crises [3]. Importantly, previous studies found that 45% of individuals who die by suicide had seen a primary care provider within a month of their death and 90% within a year of their death [4, 5]. This emphasizes the vital role that healthcare professionals can play in preventing suicide. Research further indicates that early incorporation of suicide-prevention training in medical education improves students’ intervention skills [6]. However, only a limited number of medical schools offer suicide prevention training at the preclinical stages of their curricula [7]. This gap in early medical education represents a missed opportunity to equip future physicians with the tools necessary to identify and handle suicidal ideation from the outset of their careers. To address this issue, we implemented safeTALK, a standardized suicide prevention training program, for medical students at Oakland University William Beaumont School of Medicine (OUWB). Unlike ASIST and QPR, safeTALK emphasizes recognition and referral rather than direct action. Furthermore, safeTALK was also chosen because our hospital partner, Corewell Health, had already implemented the program for its employees, providing us with existing trainers and timely delivery. This three-hour training was led by the Corewell Health Spiritual Care Team using videos, role-play, and guided discussion. Its brevity and standardized “train-the-trainer” model make it highly feasible for integration into preclinical medical education, with evidence showing sustained improvements in preparedness for up to six months after training [8]. safeTALK is a gatekeeper training program designed to teach everyday community members, such as friends, family, and educators, how to recognize warning signs of suicide and connect at-risk individuals with professional help [8]. By fostering community-wide awareness and vigilance, the program aims to create “suicide-safer communities,” empowering non-professionals to serve as early responders and support networks for those in crisis. In integrating safeTALK into the preclinical curriculum, we drew on established educational and behavioral learning theories that support the development of clinical skills. The scenario-based elements of safeTALK draw on Kolb’s Experiential Learning Theory, which highlights that active participation and reflection are critical for the acquisition of interpersonal competencies [9]. Additionally, safeTALK includes modeling and structured practice in alignment with Bandura’s Social Cognitive theory, which emphasizes the principle of self-efficacy. This allows students to develop their confidence in identifying and handling a mental health crisis, thereby improving the likelihood of effective intervention [10]. Past research by Hjelvik et al. found that even a two-hour peer-to-peer suicide prevention workshop significantly increased medical students’ self-rated knowledge and confidence for responding to a peer crisis [11]. Building on these findings, our study at OUWB broadens the scope by implementing safeTALK within the preclinical curriculum, thereby filling an important gap and enhancing the existing evidence base. Specifically, we assess the impact of safeTALK by measuring students’ self-perceived readiness in identifying and handling mental health crises before and after completing the training. By investigating the effectiveness of safeTALK within our preclinical medical curriculum, this study provides critical insight into the role of early suicide prevention training in shaping future physicians’ competency.

Evidence suggests that structured suicide prevention training programs can improve an individual’s ability to respond to suicidal behavior [6, 12]. Programs such as Tell, Ask, Listen, and Keep Safe (safeTALK), Applied Suicide Intervention Skills Training (ASIST), and Question, Persuade, Refer (QPR) have been successfully implemented [8, 13]. These kinds of trainings emphasize the value of lived experience and role-play, showing improvements in suicide prevention knowledge and confidence in asking both patients and peers about suicide.

## Methods

Kotter’s Eight-Step Change Model was used to guide the integration of a suicide prevention training for preclinical medical students [14].

### 1. Create a Sense of Urgency

We met with the M2 Behavioral Medicine and Psychopathology course director and the M3 Psychiatry clerkship director to assess existing suicide prevention instruction. We found that students received one lecture during their second year and additional exposure during their third-year Objective Structured Clinical Examination (OSCE).

To understand national practices, we distributed a survey via a group chat that included the Association of American Medical Colleges Organization of Student Representatives (AAMC OSR) from U.S. allopathic medical schools. A total of 38 OSRs responded, providing insight into the prevalence of preclinical suicide prevention training across institutions.

### 2. Build a Guiding Coalition

A multidisciplinary guiding team was assembled, consisting of medical school deans, hospital-based suicide prevention trainers, and OUWB OSR student leaders across all four classes. We also partnered with the OUWB Office of Student Affairs, who agreed to provide funding for the training.

### 3. Form a Strategic Vision

The guiding team established the goal of integrating a structured suicide prevention training program for preclinical students at OUWB. The safeTALK training was selected as the pilot initiative. A description of safeTALK is provided below.

### 4. Enlist a Volunteer Army

safeTALK was first offered as an optional session in February 2024. Information about the session was shared with students through weekly Office of Student Affairs’ email communications and class group chat texts for one month.

### 5. Enable Action by Removing Barriers

To facilitate participation, the session was scheduled during a period after examinations and before the academic break. OUWB operates across two sites, with preclinical students based at Oakland University (OU) in Rochester, MI, and clinical students based at the Corewell Health William Beaumont University Hospital in Royal Oak, MI. As the session was intended for preclinical students, we reserved a room on the OU campus to maximize attendance.

### 6. Generate Short-Term Wins

A total of 12 students, five M1s and seven M2s, participated in the pilot session and provided positive feedback.

### 7. Sustain Acceleration

**Fig. 1:**
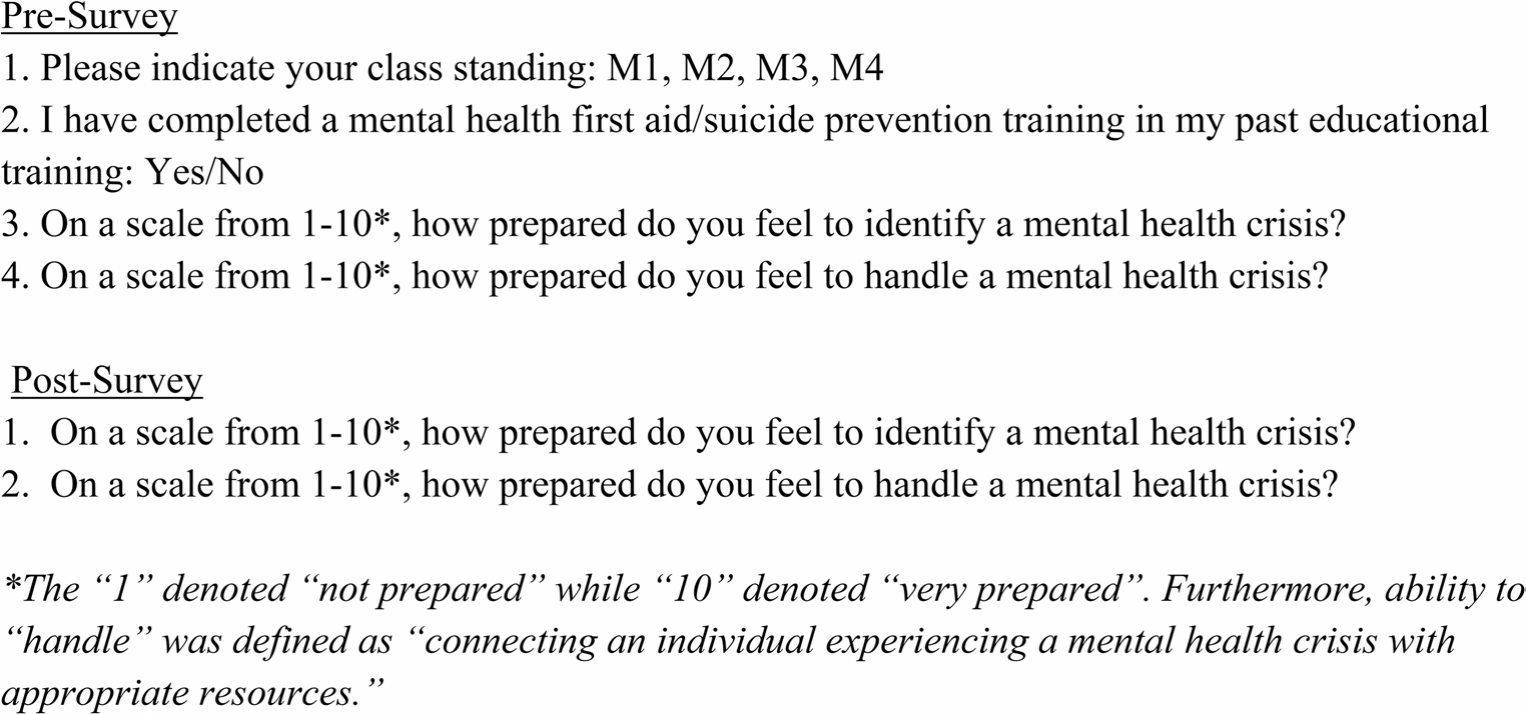
Pre- and post- surveys for the safeTALK training

Participants completed a four-question pre-training survey that assessed class year, prior suicide prevention training, and self-perceived preparedness to identify and handle a mental health crisis on a 1–10 Likert scale. A two-question post-training survey assessed changes in perceived preparedness in these same areas. The surveys are provided in Fig. 1.

### 8. Institute Change

**Fig. 2:**
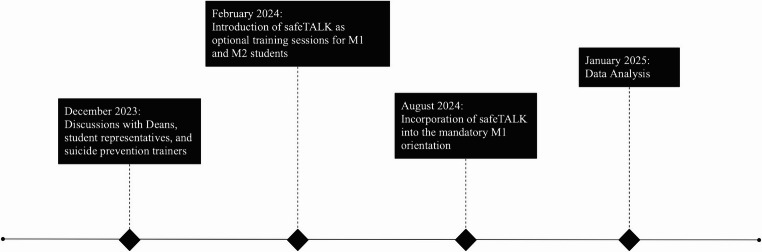
Timeline of integrating safeTALK training into the preclinical medical

Following the successful pilot, the guiding coalition agreed that, consistent with OUWB’s mission to train compassionate physicians, suicide prevention training should be offered to incoming students as early as possible. safeTALK was integrated as a mandatory session in the M1 orientation in August 2024. A total of 131 M1 students participated, with 127 completing both the pre- and post-training surveys. A timeline of events is provided in Fig. 2.

### Data Analysis

Data analysis was conducted using Microsoft Excel. A bivariate analysis comparing pre- and post-training scores was performed using paired t-tests, with statistical significance defined as *p* < 0.05. The study received approval from the Institutional Review Board (IRB).

### safeTALK Training Description [15]

#### Curriculum

safeTALK is a three-hour, interactive suicide prevention training developed by LivingWorks Education, a national organization providing evidence-based suicide intervention programs. The training equips participants to recognize signs of suicidal thoughts and respond compassionately. It introduces the TALK model or Tell, Ask, Listen, and Keep Safe, which guides engagement with individuals at risk and connects them to appropriate resources.

#### Certification

Trainers complete the two-day LivingWorks Training for Trainers (T4T) program, study standardized learning materials, and maintain accreditation through regular workshop delivery and continuous quality improvement. For this study, certified trainers from the Corewell Health Spiritual Care Team delivered safeTALK to the OUWB students.

## Results

Out of the 38 OSRs who responded to our poll, 29 (76%) reported that their medical school lacked suicide prevention training in their preclinical curriculum.

For the first session in February 2024, a total of 12 OUWB medical students (5 M1 and 7 M2) completed the safeTALK training and survey. 40% of the M1 (2/5) and 29% of the M2 (2/7) students had completed a prior suicide prevention training in their past education.

In regards to preparedness in identifying a mental health crisis, M1 students reported an average rating of 6.4, and M2 students reported an average rating of 5.7 prior to completing safeTALK. After the training, the M1 average rating went up by 2.0 (95% CI 0.5–3.5) for an average rating of 8.4 (*p* < 0.05). For the M2s, the average rating went up by 2.9 (95% CI 0.9–4.8) for an average rating of 8.6 (*p* < 0.05). For preparedness in handling a mental health crisis, M1 students reported an average rating of 5.6, and M2 students reported a rating of 5.3 prior to the completion of the training. After the training, the M1 average rating went up by 2.0 (95% CI 0.2–3.8) for an average rating of 7.6 (*p* < 0.05). The M2 average rating went up by 3.1 (95% CI 0.8–5.5) for an average rating of 8.4 (*p* < 0.05) (Table 1).

**Table 1:** Class of 2027 (M1) and class of 2026 (M2) optional session: self-perceived ability to identify and handle a mental health crisis

Class	Survey stage	Ability to identify	Ability to handle
Class of 2027 (*n* = 5)	Pre-survey	6.4	5.6
	Post-survey	8.4	7.6
	Difference (95% CI)	2.0* (0.5–3.5)	2.0* (0.2–3.8)
Class of 2026 (*n* = 7)	Pre-survey	5.7	5.3
	Post-survey	8.6	8.4
	Difference (95% CI)	2.9* (0.9–4.8)	3.1* (0.8–5.5)

*p < 0.05

For the second session in August 2024, a total of 127 incoming OUWB M1 students completed the safeTALK training and survey. 31% (39) of the incoming M1 students had completed a prior suicide prevention training in their past education. In regards to preparedness in identifying a mental health crisis, students reported an average rating of 6.2 prior to the training. After the training, this went up by 2.8 (95% CI 2.5–3.1) for an average of 9.0 (*p* < 0.001). For preparedness in handling a mental health crisis, students reported an average rating of 5.2 prior to the training. After the training, this went up by 3.4 (95% CI 3.1–3.7)for an average rating of 8.6 (*p* < 0.001) (Table 2).

**Table 2:** Class of 2028 (M1) orientation session: self-perceived ability to identify and handle a mental health crisis

Class	Survey stage	Ability to identify	Ability to handle
Class of 2028 (*n* = 127)	Pre-survey	6.2	5.2
	Post-survey	9.0	8.6
	Difference (95% CI)	2.8*** (2.5–3.1)	3.4*** (3.1–3.7)

***p < 0.001

## Discussion

Through this study we found that the majority of medical students have not completed a suicide prevention training in their prior education. Our initial survey also showed that most medical schools do not offer a suicide prevention training in their preclinical education. We first decided to pilot safeTALK as an optional training for preclinical students and had a total of 12 M1 and M2 students attend. Once we incorporated safeTALK as a part of M1 orientation, 127 participated in the training and submitted surveys.

Before adding safeTALK to M1 orientation, we had considered making it a part of our Behavioral Medicine and Psychopathology course as well as our longitudinal wellness, clinical skills, or public health courses. We decided to go with the M1 orientation as it allows us to train the greatest number of students at the earliest time possible. Evidence also shows that M1 students experience higher levels of stress than their gender-matched peers in the general US population, and these rates progressively increase throughout the academic year [16]. By including safeTALK as part of the M1 onboarding process, we wanted to proactively equip students with the skills needed to identify and handle signs of suicidality not only in their patients, but in their peers as well. Through safeTALK, we are able to cultivate a culture of mental health awareness and emphasize that the OUWB community is a safe space for students.

Although only 12 students completed the pilot session, the smaller sample size may have been influenced by the session being optional and the M1 students’ having an examination earlier that morning. We were able to successfully expand the sample size when safeTALK became a mandatory part of the M1 orientation. Students in both sessions showed a statistically significant increase in their preparednessness to manage a mental health crisis after completing the safeTALK training. Ideally, for any students who did not get the opportunity to complete the training (remaining M2, M3, and M4 students), additional safeTALK sessions can be offered as an optional or mandatory training at the beginning of their upcoming academic year or during breaks, a time when students are less burnt out and more motivated.

A key limitation of this study is that optional participants self-selected into the training, potentially reflecting higher intrinsic motivation or interest in mental health topics compared to required M1 participants. This may have introduced response bias, yet both groups still showed significant improvements in self-perceived confidence following safeTALK. Another limitation is that survey responses were collected immediately post-training, leaving the durability and practical application of these skills over time unassessed. Future studies should evaluate long-term retention, such as at the end of preclinical years or during clinical rotations. Additionally, the surveys lacked qualitative feedback; including open-ended questions in future cohorts could provide valuable insight into the training’s strengths and areas for improvement.

In terms of the training itself, one challenge was that the practice scenarios were more general than specific to medical students. Our goal is to work with training coordinators to design example scenarios that are more in line with real-life situations that medical students may encounter in the clinic and classroom. By incorporating scenarios that address mental health crises in medical students themselves, they can also be equipped with the skills to help their peers.

This study demonstrates the utility of safeTALK, a three-hour interactive suicide prevention training in preclinical medical education. Given medical students’ very busy schedules and decreased free time when they start medical school [17], we found that a short, interactive training like safeTALK being incorporated into their orientation week is more likely to fit into students’ schedules and keep them engaged. safeTALK allows students to not only learn how to identify and handle a mental health crisis, but also apply it through role play. Given the ever-increasing need for mental health support, this course provides medical students with skills that will serve them throughout their careers. Based on our study’s findings, we are advocating that safeTALK remain a mandatory part of the M1 orientation, as this delivery method allows all students to be trained as part of their medical school onboarding process. Moving forward, we would like to expand safeTALK to other medical schools and continue reducing the stigma surrounding mental health one student at a time.

## Supplementary Information

Below is the link to the electronic supplementary material.

Supplementary Material 1

Supplementary Material 2
