# Supplementary material for: Integration of Suicide Prevention Training into the Preclinical Medical School Curriculum
Source: Med Sci Educ. 2026 Mar 3;36(3):1353–8. doi: 10.1007/s40670-026-02637-3 (PMC13356117; doi:10.1007/s40670-026-02637-3)
Supplement: Supplementary file 2 — Supplementary Material 2 [file 40670_2026_2637_MOESM2_ESM.docx]

***Medical Science Educator* Reviewer Comments & Author Responses**

**“Integration of Suicide Prevention Training into the Preclinical Medical School Curriculum”**

We are grateful to the associate editor and reviewers for their insightful and helpful comments. We appreciate the time and care taken in evaluating our work. We have addressed each point in detail below and revised the manuscript to strengthen clarity, rigor, and contribution to the literature.

***As of December 28, 2025:***

| **Associate Editor Comments** | **Response** |
| --- | --- |
| Abstract and introduction: In the abstract data cited from ref (1) stated the year as "2021." In the introduction, the same reference year was "2022." Please double check and align the 2 dates. | Thank you for this important suggestion. The website continued to update over the period of time that we worked on this manuscript. As of March 2025, data from the CDC shows that there were approximately 1.5 suicide attempts in 2023, which is the statistic we have now updated in both the introduction and abstract. |
| Results: For all data in the discussion and tables (table 1, 2) with p-values, please also include the confidence intervals associated with all p-values. | We have revised the Results section and updated Tables 1 and 2 to include the corresponding confidence intervals for all reported p-values. The confidence intervals are now presented alongside the effect estimates to improve clarity and interpretability of the results. Thank you for this suggestion. |
| Acknowledgement: Please clarify "Ven." Consider providing the full spelling. | Thank you for this suggestion. We have revised the text to replace the abbreviation “Ven.” with the fully spelled title “the Venerable.” |

***Previous Comments & Responses (Submitted December 8, 2025)***

| **Reviewer 1 Comments** | **Response** |
| --- | --- |
| Feedback on the introduction/background/rationale/theoretical framework:  Well described study that identifies an important area of interest for medical education that is unfortunately not covered in many school's curriculum. | Thank you for this positive feedback. We appreciate your assessment of the study’s importance and clarity. |
| Feedback on the results/discussion or main body of the manuscript:  Pretty well done. Simple and to the point, and that makes the results clear and impactful. | Thank you for your encouraging comments regarding the results and discussion. We are glad the presentation was clear and impactful. |
| Feedback on the conclusions/summary:  The conclusion is clear: not providing teaching or training in suicide awareness or how to handle an emergent situation is a lost opportunity. The data show that students receiving this training at OUWB benefit from the training. This can be a good model for other schools seeking ways to incorporate suicide prevention into their curriculum. | We appreciate your positive evaluation of the conclusion and its relevance for broader medical education efforts. |
| Feedback on the appropriateness and timeliness of the reference list:  Good list. | Thank you for reviewing the reference list and finding it appropriate and current. |
| Feedback on the tables and figures: Figure 2 may not be necessary since the text stated the distribution. | Thank you for this suggestion. After considering your comment, we have removed Figure 2 to avoid redundancy, as the distribution is already clearly described in the text. |

| **Reviewer 2 Comments** | **Response** |
| --- | --- |
| Recommendations for Figures and Tables  The manuscript's visual presentation can be significantly improved by converting several figures into tables and consolidating data reporting into the main text. | Thank you for this thoughtful recommendation. We agree that a tabular format is more appropriate for presenting these discrete pre- and post-intervention values. In accordance with your suggestion, we have converted Figures 3 and 4 into Table 1 and Table 2, respectively. This revision improves clarity, reduces redundancy, and enhances the overall readability of the manuscript. |
| Conversion to Tables (Figures 3 and 4) to tabular data  I recommend that Figures 3 and 4 be converted to tables. The current bar chart format does not enhance reader comprehension or visually emphasize a powerful trend, thereby consuming valuable print space inefficiently.  Table 1: Should present the results from the initial, smaller study group of M1 and M2 students (currently Figure 3).  Table 2: Should present the results from the larger M1 cohort from the subsequent academic year (currently Figure 4).  Figures, such as bar charts, scatter plots, or line graphs, should be reserved for instances where:  1. There are a high volume of data points that are most clearly visualized on an XY plot or similar schema.  2. A meaningful trend or relationship in the data is best represented graphically for immediate visual impact.  Given that the values in Figures 3 and 4 simply represent pre- and post-intervention scores, a tabular format is the most appropriate and effective method for displaying these discrete numerical results. | In accordance with your suggestion, we have converted Figures 3 and 4 into Table 1 and Table 2, respectively. Thank you for your thorough recommendations! |
| Omission of Pie Chart (Figure 2)  The pie chart for Figure 2 should be omitted entirely. The simple count or proportion of medical schools with suicide prevention training programs is already reported in the text of the results section. Similar to the rationale above, this basic graphic does not add any interpretive value beyond simply stating the numerical values, making it redundant and unnecessary. | After considering your comment as well as the first reviewer’s similar comment, we have removed Figure 2 to avoid redundancy, as the distribution is already clearly described in the text. Thank you for this helpful observation. |
| Positive Manuscript Elements  I appreciate the inclusion of the actual questionnaire item(s) and the timeline of the project. Both additions are highly appropriate and helpful for readers seeking to understand the study's methodology and context | Thank you for your positive feedback. We are glad that the inclusion of the questionnaire items and project timeline enhanced the clarity and methodological transparency of the manuscript. |

| **Reviewer 3 Comments** | **Response** |
| --- | --- |
| Feedback on the introduction/background/rationale/theoretical framework:  The introduction effectively establishes the significance and need for suicide prevention and identifies an objectively clear gap in UME curriculum. The authors effectively identify the public health need and provide a rationale for early intervention. The literature review could be strengthened by including comparative analysis of different programs and methods in health professions or health professional education. | Thank you for the constructive feedback. We appreciate your recognition of the introduction’s clarity and rationale. In response to your recommendation, we have expanded the literature review to include a comparative discussion of suicide prevention training programs such as safeTALK, QPR, ASIST, and other instructional methods used in health professions education. This addition strengthens the contextual foundation and more clearly situates our initiative of using the safeTALK training within the broader landscape of existing approaches. |
| For Research Articles: Feedback on the design, methods and statistics:  The pre and post-survey design is appropriate for program eval and the statistical methodology sound. Authors could consider expanding discussion on sampling methods for the OSR participants and summarize characteristics of the optional attendees related to potential response bias in the required versus optional participants. | In response to this suggestion, we have expanded the Discussion section to include additional detail about the sampling approach for OSR participants and the potential implications of including both required and optional attendees in the evaluation. Specifically, we now summarize the potential characteristics of the optional attendees and address how their voluntary participation may introduce response bias compared to the required participants. We agree that these additions strengthen the manuscript by providing clearer context for the interpretation of our pre- and post-survey findings. Thank you for the feedback! |
| Feedback on the results/discussion or main body of the manuscript:  The results are clearly presented with simple yet effective data visuals. The discussion adds context to the findings and addresses limitations of the study. The discussion on M1 voluntary outcomes’ lack of statistical significance can be expanded beyond sample size, including the timing of the offerings and confounding variables related to participants. Authors should also consider expanding the discussion to include voluntary versus mandatory participation and how that might affect engagement and outcomes. The authors' inclusion of practical insights related to implementation timing, student stress, and limitations of the program's curriculum strengthen the discussion and applicability to the field. | Thank you for this suggestion. We have expanded the Discussion to further explore the lack of statistical significance in the M1 voluntary outcomes, going beyond sample size to address timing of the sessions and potential confounding variables among participants. We also elaborated on the voluntary versus mandatory participation and the ways these differing contexts may impact motivation, attendance, and survey responses. We believe this revision strengthens the overall interpretation and applicability of our findings. |
| Feedback on the conclusions/summary:  Conclusions are strong and accurately reflect the study's findings and provide actionable recommendations for integration in other programs. The authors find an appropriate balance between data-driven advocacy and objective reporting of findings. The self-reporting and immediacy of survey are limitations, and the authors effectively address the timing, though could more directly address the self-reported nature of the findings. | Thank you for the positive feedback and suggestions. We revised the Discussion to more directly address the limitation related to the self-reported nature of our data, especially with students in the initial study who volunteered for the training. We believe these additions further strengthen the transparency and strength of our conclusions. |
| Feedback on the appropriateness and timeliness of the reference list:  References are current and relevant. References to educational theory on skills development (timing, redundancy) would strengthen the theoretical basis. | Thank you for this very helpful suggestion. In response, we have expanded the Introduction to include a paragraph discussing Kolb’s Experiential Learning Theory and Bandura’s Social Cognitive Theory to further explain the theoretical framework supporting early skills development in suicide prevention training. These additions strengthen the rationale for incorporating programs such as safeTALK into the undergraduate medical curriculum. |

| **Associate Editor Comments** | **Response** |
| --- | --- |
| Consider adding the following reference to the introduction: "A Peer-to-Peer Suicide Prevention Workshop for Medical Students" | We reviewed the suggested reference and agree that it is highly relevant to the context of our work. We have incorporated this citation into the Introduction to further strengthen the background and highlight comparable suicide prevention initiatives within medical education. Thank you for this valuable recommendation. |
| Elaborate on the safeTALK training - what is the content, and does the training entail? | Thank you for this helpful suggestion. In response, we have expanded the Methods section to provide a clearer and much more thorough description of the safeTALK curriculum. The revised text outlines the core components of the training, including the recognition of suicide warning signs, use of the “Tell, Ask, Listen, KeepSafe” (TALK) framework and engagement in guided practice activities. We agree that this added detail enhances readers’ understanding of the program’s content and instructional approach. |
| What other suicide prevention training programs are available? Why was safeTALK selected over other programs? | Thank you for raising this important point. In response, we expanded the Introduction to briefly review other widely used suicide prevention training programs, including ASIST and QPR, and summarized comparative findings from existing studies. We also added clarification regarding our selection of safeTALK, noting its suitability for brief, skills-focused training within a preclinical curriculum, its strong evidence base, and its emphasis on practical, actionable frameworks for identifying and responding to individuals at risk. These additions provide clearer justification for the choice of safeTALK over alternative programs. |
| Include literature review that supports the effectiveness of safeTALK in preventing suicide. How was the program developed and what theoretical frameworks supported its development and use? | Thank you for this important suggestion. In response, we have expanded the Introduction to include a focused literature review supporting safeTALK’s effectiveness, including longitudinal evidence from Holmes et al. documenting sustained improvements following training. We also framed the program’s effectiveness in the Introduction within established educational and behavioral theories (Kolb’s Experiential Learning Theory and Bandura’s Social Cognitive Theory). Lastly, we expanded on the Methods section to add a brief description of safeTALK’s development and a detailed description of the training. These additions provide a more clear rationale for incorporating safeTALk in the medical curriculum as a skills-based suicide prevention training. |
| Include the certification process for safeTALK trainers - this is important if other schools want to implement the program. | Thank you for highlighting the importance of trainer certification for program implementation at other schools. In response, we have added a description of the safeTALK trainer certification process to the Methods section, outlining the requirements for becoming a certified safeTALK facilitator. This addition supports implementation feasibility for other schools interested in adopting the program. |
| It would be invaluable to readers to also understand the authors' experience in implementing this change to the curriculum - what was the inspiration? What framework was used to guide the change? (eg. Kern's, Kotter etc). What did the collaboration process look like? | Thank you for your feedback. We have added to our Methods section, describing our experience implementing the curriculum change. This includes the inspiration behind the project, our step-by-step use of Kotter’s Eight-Step Change Model to guide development and integration, and the collaborative process involving faculty, student representatives, and mental health professionals. We believe this provides readers with valuable insight into both the rationale and practical considerations behind the curriculum implementation. |
