# Supplementary material for: Integration of Suicide Prevention Training into the Preclinical Medical School Curriculum
Source: Med Sci Educ. 2026 Mar 3;36(3):1353–8. doi: 10.1007/s40670-026-02637-3 (PMC13356117; doi:10.1007/s40670-026-02637-3)
Supplement: Supplementary file 1 — Supplementary Material 1 [file 40670_2026_2637_MOESM1_ESM.pdf]

Ahmad Abu-Mahfouz
Oakland University William Beaumont School of Medicine
abumahfouz@oakland.edu
December 29, 2025

Dr. Khiet DT Ngo
Associate Editor
Medical Science Educator

Dear Dr. Ngo,

Thank you again for the opportunity to revise and resubmit our manuscript for consideration in *Medical Science Educator*. During our final review of the manuscript, we made a few adjustments in accordance to your comments as well as a few additional ones:

1. **Statistical analysis correction (Table 1):** For the M1 ability to *handle* a crisis in Table 1, the p-value from a two-sample t test was listed instead of the paired t-test p value. We re-examined this and inputted the correct p-value into Table 1, which showed significance. The corresponding text in the Results and Discussion sections have also been updated to reflect this change. This strengthens the evidence for the effectiveness of the intervention and does not affect any other findings or conclusions of the manuscript.
2. **Confidence intervals for Table 1 and Table 2:** As suggested, we added 95% confidence intervals for all the associated p-values in the Results, as well as in Table 1 and Table 2.
3. **Reference year and CDC data update (Abstract and Introduction):** The CDC website has continued updating their statistics regarding suicide over the period of our manuscript preparation. As of March 2025, their data indicates approximately 1.5 million suicide attempts in 2023. We updated both the Abstract and Introduction to reflect this current statistic.
4. **Acknowledgement clarification:** We revised the text to replace the abbreviation “Ven.” with the fully spelled title “the Venerable,” as suggested.

The revised manuscript is submitted with all these corrections incorporated. We appreciate your understanding of these final updates. Thank you for your time and for considering us for publication in *Medical Science Educator*.

Sincerely,

Ahmad Abu-Mahfouz (on behalf of all co-authors)
Oakland University William Beaumont School of Medicine
abumahfouz@oakland.edu
